# 688. Risk of *Clostridioides difficile* Infection and Antibiotic Associated Diarrhea in Patients Receiving Outpatient Parenteral Antimicrobial Therapy

**DOI:** 10.1093/ofid/ofad500.750

**Published:** 2023-11-27

**Authors:** Mario Jaramillo, Ahmad S Musmar, Aishan Shi, Shaden Al Momani, D Alexander Perry, Tirdad Zangeneh

**Affiliations:** University of Arizona-Tucson, Tucson, Arizona; University of Arizona, Tucson, Arizona; Banner University of Arizona Tucson, Tucson, Arizona; University of Arizona-Tucson, Tucson, Arizona; University of Arizona-Tucson, Tucson, Arizona; University of Arizona, Tucson, Arizona

## Abstract

**Background:**

The colonic microbiome plays a critical role in resisting the ingrowth of pathogenic microbes like *Clostridioides difficile (C. difficile)*. Disruption of the microbiome by antibiotics is known to cause antibiotic-associated diarrhea (AAD) and *C. difficile* infection (CDI). Outpatient parenteral antimicrobial therapy (OPAT) reduces hospital length of stay and exposure to healthcare-associated pathogens which may decrease the incidence of CDI. Previous studies that compared patients who received metronidazole for non-CDI indications with those who did not found a reduction in CDI among patients who received metronidazole. The aim was to study the incidence of AAD and CDI in the OPAT patient population and compare the incidence rate between patients who received oral metronidazole for non-CDI indications with those who did not.

**Methods:**

We retrospectively reviewed a cohort from our facility's OPAT program. We included adult patients (18 years or older) discharged to home or skilled nursing facility (SNF) on OPAT over a 2-year time span, regardless of diagnosis. Pregnant patients, patients with active CDI before starting OPAT, and those who were receiving an associated CDI treatment were excluded. Antibiotic-associated diarrhea was defined as 3 or more loose or watery stools per day with negative CDI testing or spontaneous improvement after stopping antibiotics. Community-onset and healthcare facility–associated CDI (CO-HCFA CDI) was defined as 3 or more watery stools per day with a positive stool *C. difficile* diagnostic test in the community or within the first 3 days of readmission, provided the diagnosis was made less than 4 weeks since discharge from a healthcare facility.

Demographics of Patients Discharged on OPAT.

**Figure:**
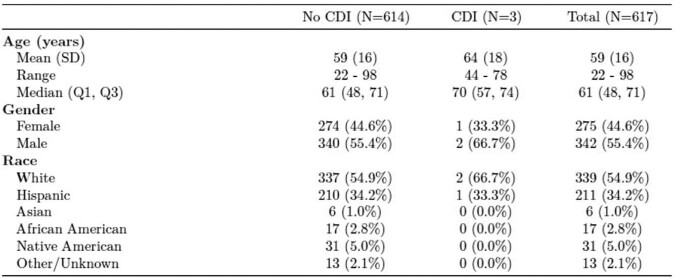

Patients selected between June 1, 2018 and June 2020, grouped by whether or not they developed Clostridioides difficile infection (CDI). SD= standard deviation; Q1= 1st quartile; Q3= 3rd quartile.

**Results:**

Of the 617 charts that met inclusion criteria, 31 had AAD and 3 had CO-HCFA CDI. Incidence of CO-HCFA CDI was < 1%; < 1 per 1000 person-day and incidence of AAD was 5.02%. The protective role of metronidazole could not be studied due to the rarity of CDI in this study.

Patients with positive CDI after initiation of OPAT and associated characteristics.

**Figure:**
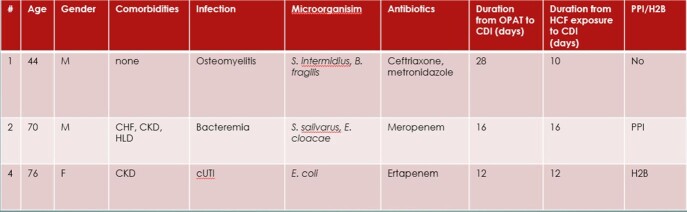

**Conclusion:**

Although our study was not able to answer our initial question when comparing the effect of metronidazole within OPAT groups and rates of CDI, our data is promising for showing low rates of CDI in patients receiving OPAT. This study demonstrates the putative benefits of OPAT with reduced CDI incidence regardless of use of anti-CDI drugs.

**Disclosures:**

**Tirdad Zangeneh, DO**, AiCuris: Grant/Research Support

